# Progress in Surgery

**Published:** 1896-06-27

**Authors:** 


### PROGRESS IN SURGERY.

### GENITO-URINARY SURGERY.

(*Continued from page* 178.)

**Tumours and Pouches of the Bladder.**—Mr. Charles A. Morton[1] records a case of pedunculated papilloma of the bladder removed by scissors through a suprapubic opening, in which severe secondary hæmorrhage occurred on the third day. Scissors are now frequently used for cutting through the base of the growth if pedunculated, and there was no trouble from hæmorrhage at the time of the operation, firm sponge pressure being quite sufficient to stop all bleeding. An interesting and instructive point in the case was a collection of clots outside the bladder, between it and the anterior abdominal wall, just behind the pubes, and around the tube through which urine was draining away from the bladder. The skin incision had been sutured around the tube. There was nothing to distinguish this condition from a collection of clots within the bladder. He thinks the contraction of the bladder from time to time broke down the sutures and forced the clots out into the prevesical space. Washing out the bladder with very hot boracic lotion with a Bigelow's apparatus stopped the hæmorrhage and removed some clot; but the bleeding recurring, the suprapubic wound was reopened, the extra vesical collection of clot discovered and turned out, and then perchloride of iron applied to the stump of the pedicle. The patient had no further hæmorrhage, and made a satisfactory recovery. The growth had acted as a ball-valve in the neck of the bladder, and caused great pain and sudden stoppage of micturition, as well as frequent hæmaturia. The question of drainage of the bladder is discussed, and Mr. Morton refers to a case of suprapubic lithotomy for large calculus, in which, although marked cystitis was present, he obtained primary union by suturing the bladder opening, and he thinks this may always be safely done if at the same time the surgeon drains the space between the sutured bladder and the sutured abdominal wall, for then, if the sutures give way, he is no worse off than if he left the bladder open from the first. But as the cystitis persisted after the lithotomy in that case he does not feel sure that it would not have been better to have drained the bladder from above the pubes, as in the case of bladder tumour he records.

Mr. Targett showed at the Pathological Society[2] two specimens of cystic tumours associated with malignant disease of the bladder. In one there was an ordinary bladder sacculus, which had become invaded by epithelioma, but in the other the cystic cavity was formed not by the bladder wall but by the recto-vesical sheath. This had been distended by extravasated urine which had escaped through a perforation made by ulceration of the malignant growth in the vesical wall. The left ureter, vas deferens, and vesicula seminalis and peritoneum were stripped off the bladder by the enlargement of the cyst. Mr. Henry Fenwick[3] also records a case in which a swelling the size of an ostrich's egg formed suddenly just above the pubes, and remained unaltered in volume after the evacuation of the bladder by catheter. This seemed to preclude the idea of its being a diverticulum, but the autopsy showed it to be of this nature, the orifice being secluded by an epitheliomatous growth.

Reginald Harrison, at a clinical lecture at St. Peter's Hospital,[4] describes various cases of sacculation of the bladder. He agrees with Mr. Targett that sacculation may be congenital, as the result either of intra-uterine pressure or of developmental variations, but the most frequent cause he believes to be obstruction from the prostate or in the urethra. He relates one case which he considers to be of traumatic origin. These saccules (which are formed by a protrusion of the mucous membrane between the muscular layers of the bladder wall) are particularly serious if lithotrity is practiced, as the fragments fall into them and remain within, thus setting up severe cystitis. There seem to be no distinct signs of their presence, but in some cases we may diagnose them by the fact that a catheter empties two distinct collections of urine, possibly differing in character, and a case is recorded where a patient did not feel comfortable after passing urine until by pressing on the inner side of the left tuberosity of the ischium he got rid of two or three ounces more. In other cases the sacculus forms a hypogastric swelling, which can be dispersed by the use of the catheter. Occasionally they are found in hernial sacs as extra-peritoneal diverticula from the bladder, and have now and again been cut off under the mistake that they were the sac. Mr. Harrison believes that if we can diagnose a sacculus as a complication of chronic cystitis it is an additional reason for suprapubic drainage of the bladder. He hints at the possibility of excising some sacculi, as they have been cut off in hernia[1] sacs without bad results, and suggests that in cases in which operation is not called for, the constant movement afforded by a sea voyage may keep the sacculus emptied and the patient less liable to cystitis.

As the result of a careful study of the recorded cases of myomata of the bladder the following conclusions have been arrived at.[5] They are in many respects analogous to uterine fibro-myomata, and take their origin in the muscular layer of the bladder, projecting upon either its external or internal surface, most frequently into the organ. If they develop beneath the connective tissue of the pelvis they are sessile; they tend to become pedunculated if they grow into the peritoneal cavity. They are frequently encapsuled, may calcify or slough. They have been removed under the impression they were myomata of the uterus. When the cavity of the bladder is invaded, hæmaturia (as in papilloma) is one of the most frequent symptoms, and later cystitis may set in. Hypogastric, vaginal, or rectal palpation may discover the growth, especially if bimanual. Some of the growths which project outside the bladder may attain a large size, obliterating the prevesical space and raising the peritoneum, As they are generally encapsuled when growing from the outside of the bladder their enucleation is recommended, the wound in the bladder wall being closed by suture. The intra-vesical growths should be removed in the same way by suprapubic cystotomy, the écraseure and galvanic cautery being unnecessary.

**Intraperitoneal Suprapubic Lithotomy.**—Maurice H. Richardson[6] records two cases. The operation was intraperitoneal, because the prevesical space was obliterated, the peritoneum passing down behind the bladder which was too contracted to be distended. The stones were very large in both cases, and could not be dealt with by lithotrity, though the attempt to grasp the stones was made. In the first case the fundus was temporarily sutured to the middle of the abdominal incision, and the abdominal cavity above well packed with sterilised gauze. In the second the suturing was not possible, but the abdominal cavity around was thoroughly packed with gauze. In both the bladder was kept empty after the operation by being packed with gauze. No peritonitis occurred in either, both patients making a satisfactory recovery. So contracted was the bladder in one of the cases that, instead of distending as fluid was injected into it, the fluid passed between the coats and collected under the peritoneum, the muscular coat contracting firmly around the stone. Reference is made by Richardson to a paper by Harrington, of Boston, "On the Feasibility of Intraperitoneal Cystotomy," with the report of a case.

**Prevesical Inflammation.** — A case in which the bladder would not hold more than two ounces, and hence could not be distended for suprapubic cystotomy is recorded by Hotchkiss,[7] of New York. The patient suffered from calculus and chronic cystitis, and there was extreme tenderness above the pubes, and the abdominal wall in that region seemed hard, dense, and unyielding, and here on operation a dense mass of fibroid tissue was discovered, on cutting through which the bladder was entered before its walls could be recognised. At the autopsy general perivesical inflammatory adhesions were found, particularly dense in the prevesical space. Dr. Ernest Michels[8] records four cases of prevesical abscess, and points out the difficulty which may arise in distinguishing such collection of pus from solid growths. They usually produce a hypogastric swelling and frequency of micturition, but the urine is normal. They are generally due to disease of pelvic structures other than the bladder, chiefly the pelvic bones. The abscess may spread in the pelvis or open into one of the pelvic organs.

**Intraperitoneal Rupture of the Bladder.**—A successful case of suture is recorded by Heaton, of Birmingham.[9] The operation was performed fifteen hours after the accident. The case is one of those rare instances of ruptured bladder in women. The rent measured three and a half inches in length. It was closed by a row of sutures uniting the serous and muscular coats, and then another turning in the same coats, as in Lambert's suture. The bladder was then injected, and found not to leak; the abdominal cavity was flushed out with water sterilised by boiling, and the abdominal incision closed without drainage. The urine was drawn off for three weeks. Attention is called to the fact that there may be no shock in these cases of abdominal injury, and no discoloration of the skin, the patient walking up to the hospital, perhaps, with the complaint that it is impossible to make water. Of thirty-four cases of intraperitoneal rupture of the bladder treated by suture, fourteen recovered and twenty died.

[1] Lancet, Feb. 22, 1896, p. 480. [2] Lancet, March 21, 1896, p. 766. [3] Ibid. [4] International Clinics, vol. iv., p. 243. [5] Annals of Surgery, Feb., 1896, p. 221. [6] Annals of Surgery, Feb., 1896, p 132. [7] Ibid, p. 141. [8] Lancet, Jan. 18, 1896. [9] Lancet, April 11, 1896, p. 991.

